# Ambulatory Blood Pressure Monitoring in a Cohort of Children Referred with Suspected Hypertension: Characteristics of Children with and without Attention Deficit Hyperactivity Disorder

**DOI:** 10.1155/2013/419208

**Published:** 2013-07-22

**Authors:** Silviu Grisaru, Melissa W. Yue, Justin C. Mah, Lorraine A. Hamiwka

**Affiliations:** ^1^Alberta Children's Hospital, Division of Paediatric Nephrology, University of Calgary, 2888 Shaganappi Trail NW, Calgary, AB, Canada; ^2^The O'Brien Centre for the Bachelor of Health Sciences Program (BHSc), University of Calgary, Calgary, AB, Canada

## Abstract

Childhood hypertension's increasing prevalence has generally been linked to the obesity epidemic. We observed that a significant proportion of children referred to our pediatric center with documented office hypertension are nonobese and have a history of attention deficit hyperactivity disorder (ADHD). To define the extent of this anecdotal observation, we performed a retrospective analysis of ambulatory blood pressure monitoring (ABPM) tests which in our center are routinely performed in newly referred children suspected of hypertension. Twenty-one percent (48 of 227 new referrals) had a history of ADHD, and 81% of them were treated with psychostimulant medications at the time of their ABPM test. Children in this group had a significantly lower average BMI *z*-score compared with the rest of the children (0.18 versus 0.75) and were significantly more likely to have abnormally elevated wake systolic loads on ABPM (38% versus 4%). The overall proportion of children with any abnormality on ABPM was comparable in both groups (46% versus 40%). *Conclusion*. A significant proportion of children suspected of hypertension have ADHD which may be related to higher wake systolic BP values. The prevalence of hypertension among children with ADHD will have to be determined in prospective studies.

## 1. Introduction

The prevalence of childhood hypertension has been rising steadily, and the obesity epidemics have largely been blamed for this trend [1, 2]. Increased awareness, better diagnostic criteria, and new tools such as ambulatory monitoring have also been credited for the growing number of children diagnosed with hypertension or prehypertension [3]. 

The Pediatric Nephrology Clinic in our tertiary care pediatric centre receives referrals from physicians in the community to further investigate and manage children suspected of hypertension. Since 2003 most of these children aged 6 years and older are screened with a 24 h ambulatory blood pressure monitoring (ABPM) test prior to their first clinic visit. In addition, new referrals are triaged for priority by clinic physicians during routine weekly division meetings. This triage process led to the anecdotal observation that a significant proportion of children referred for suspected hypertension had attention deficit hyperactivity disorder ADHD mentioned in the referral as background comorbidity. 

Behavioral abnormalities and ADHD have recently been shown to be associated with hypertension in pediatric patients [4, 5]. In addition, stimulant agents which are the most common type of treatment for ADHD have been shown to cause a modest but consistent increase in heart rate and blood pressure values [6]. These changes have mostly been considered as clinically insignificant [6, 7]. Nevertheless, long-term implications associated with prolonged exposure to these changes as well as potentially increased risk in subgroups of children with other comorbidities are currently ill defined.

In view of our observations we sought to define the prevalence of ADHD among children referred to our clinic for suspected hypertension and compare their baseline BMI as well as ambulatory blood pressure monitoring results to children referred for similar reasons without a history of ADHD. 

## 2. Methods

Our center serves a population of roughly 1.5 million inhabitants. As mentioned previously the vast majority of children newly referred for evaluation of suspected hypertension are routinely screened with an ABPM test prior to their first visit. When tests are performed, patients are measured and weighed by the nurse administering the test who also records the following information: indication for the test, referring physician, previous medical history, and prescribed medications. These data as well as patients' age and gender are included in the final reports of all ABPM tests. After review by the local Research Ethics Board waiving the need for signed informed consent, we examined all ABPM test reports performed at our centre from 2005 to 2010. Screening ABPM tests performed prior to the first visit of newly referred patients were included. Follow-up ABPM tests for patients with known hypertension or kidney disease were excluded. All available data from the tests' reports were collected and entered in an electronic spreadsheet. 

All patients included in this study were referred by community pediatricians or family physicians who performed office screening blood pressure measurements which were noted to be higher than the 95th percentile for age height and gender. There was no data available on the number of occasions on which this was documented. All included ABPM tests were performed as screening prior to the first visit in the Pediatric Nephrology Clinic, before any type of interventions, including stopping medications, was suggested. 

The Welch Allyn ABPM 6100 monitor was used in all cases with set parameters according to reference values provided by Wuhl et al. [8]. ABPM tests' results were interpreted according to Urbina et al. [9]. Systolic or diastolic loads defined as the percent of readings above the 95th percentile were considered abnormal if greater than 25% [9]. BMI and BMI *z*-score were calculated based on height and weight recorded at the time of the test. Statistical analyses including descriptive statistics, chi-square test, and unpaired *t*-test were performed with SigmaStat.

## 3. Results and Discussion

A total of 1158 ABPM tests were performed in 580 children. After excluding follow-up and routine tests for patients with known hypertension, acute or chronic kidney disease, and kidney transplant recipients, 227 ABPM tests performed in 227 children referred for suspected hypertension were identified. Forty-eight of them (21%) had a background diagnosis of ADHD, and 39 of these children were taking psychostimulant agents at the time of their ABPM test. The majority (31 patients) were treated with slow/sustained release methylphenidate. The rest were taking dextroamphetamine alone or in combination with amphetamine; 3 patients were on two psychostimulant medications, and 8 patients were concomitantly treated with clonidine. Among the rest of the children with ADHD, 5 were taking atomoxetine and 4 were on no drug therapy. Other prescription medications recorded as being taken regularly at the time of the ABPM test included the following.

- Among patients with ADHD, 3 were taking melatonin, 2 risperidone, 1 flunarizine, and 1 insulin.
- Among patients without ADHD, 9 were treated with insulin, 7 prophylactic antibiotics, 4 salbutamol, 3 montelukast, 3 fluticasone, 2 budesonside, 2 isotretinoin, 2 ranitidine, and 2 oral contraceptives.

Patients' characteristics and ABPM results retrieved from the test reports are summarized in Table 1. 

This retrospective analysis was initiated by an anecdotal observation of a high prevalence of ADHD among children suspected of hypertension referred to our centre by community physicians. The analysis revealed that indeed in our clinic more than twenty percent of newly referred children suspected of hypertension had a history of ADHD. However, the validity of this observation is likely to have been affected by the study's methodological limitations, most important of which would be referral bias. For example, all children with ADHD that were included in this study were referred by community pediatricians rather than family physicians, and children with Type 1 diabetes, as deducted by the number of patients treated with insulin, were significantly overrepresented in our cohort. These observations suggest that the initial office blood pressure screening may have been targeted to children with certain comorbidities rather than the general population. Nevertheless the incidence of hypertension among children with ADHD has never been established, and while this retrospective analysis cannot fill this knowledge gap, it highlights the need for properly designed studies aimed at establishing the burden of hypertension and prehypertension among children with ADHD.

The blood pressure standards published in The Fourth Report on the Diagnosis, Evaluation, and Treatment of High Blood Pressure in Children and Adolescents [10] are now widely used by clinicians for screening and diagnosis of hypertension in pediatric patients; however, ambulatory blood pressure monitoring is becoming increasingly available and routinely used. Ambulatory blood pressure monitoring helps to identify “white coat” and “masked hypertension” as well as facilitates followup of children with prehypertension and hypertension [9]. In addition, ABPM has also been shown to be better correlated with hypertension induced target organ changes such as increased left ventricular mass [9]. Routine ABPM screening of newly referred patients prior to first encounter was introduced in our clinic in 2003. In our center, test results are never used to decline referred patients; they facilitate the triage process and help identify children that are likely to exhibit office (“white coat”) hypertension. Sixty percent of the referred children in our cohort had ABPM screening tests that were interpreted as normal suggesting that they may have office or “white coat” hypertension. While this designation is not considered to be entirely benign and followup for these children is recommended, having the information available at the first visit helps to alleviate parents' concerns and allows focusing on healthy lifestyle counseling.

Careful examination of the abnormal ABPM studies in children with a history of ADHD from our cohort led to the additional observation that these children were more likely to exhibit abnormal systolic loads during the day. In fact most of the ADHD children with any type of abnormality (18/22, 82%) had wake systolic loads greater than 25%, whereas among the rest of the children this was the least common abnormality (7/71, 10%). Sleep systolic loads were rarely found to be abnormal among children with ADHD 3/22 (14%), while among the rest of the children this abnormality was significantly more common (45/71, 63%). The currently accepted standards for interpretation of ABPM tests are based on screening a cohort of healthy children and youth, accounting therefore for the circadian and sleep pattern changes associated with growth and development [8]. Attention deficit and hyperactivity disorder has generally been associated with sleep abnormalities, including more frequent and prolonged night waking periods [11]. This would lead to the expectation that children with ADHD may be more prone to demonstrate abnormal sleep blood pressure dipping on ABPM. In our cohort though, we observed an opposite tendency, despite the fact that sleep disturbances may have been common as suggested by the use of melatonin by 3 of the children with ADHD. It is tempting to associate the observed BP pattern with the administration of stimulant agents for the management of ADHD which are usually given during the day to improve school performance. However, in this retrospective analysis we lacked important details such as the criteria for diagnosis and treatment of ADHD as well as dosage and timing of medication. In addition, cotreatment with clonidine in 8 of the children taking psychostimulants further complicates interpretation of our observations. Nevertheless we documented that at the time of the ABPM test 81% of the children with a history of ADHD were taking psychostimulant agents. 

The effect of psychostimulants on blood pressure and heart rate, a small increase in both, is well documented in the literature and generally considered to be clinically insignificant [4, 6]. Risk for severe cardiovascular events such as sudden death has long been suspected to be influenced by treatment with psychostimulants; however, recently published data from a large population based study was reassuring and did not support this suspicion [12]. Nevertheless, the long-term impact of prolonged psychostimulant treatment beginning in early childhood on health in general and cardiovascular health in particular is not well defined [13]. 

In addition to having a distinct ABPM pattern, we observed that children with a history of ADHD in our cohort had a significantly lower BMI *z*-score compared to the rest of the children referred for suspected hypertension. Elevated rates of obesity have been observed in adults, adolescents, and young children with ADHD; however, in children medicated with psychostimulants obesity may be masked by the appetite suppressing effect of these medications [14]. Unmedicated children with ADHD were found over two times more likely to be overweight than children who were not on medication [14]. In our cohort most children with ADHD were medicated with psychostimulants which may be the cause for the observed lower BMI *z*-score in this group. Interestingly, the proportion of children exhibiting abnormalities on ABPM tests was comparable between the group of children with ADHD and the rest of the children despite the difference in BMI *z*-score. This observation highlights the importance of blood pressure screening in all children. Childhood obesity's strong correlation with hypertension is supported by a large body of evidence and has been emphasized in many original and review articles [1–3, 15]. The results of this retrospective analysis demonstrate that other groups of children with different comorbidities such as ADHD despite having normal or low BMI may be as likely to exhibit blood pressure abnormalities and therefore should not be overlooked with regard to blood pressure screening. Current recommendations suggest to measure blood pressure in all children at every primary care medical encounter beginning at the age of 3 years [15]. 

## 4. Conclusions

In a cohort of children referred to the nephrology clinic by primary care physicians for evaluation of suspected hypertension, a significant proportion had a documented history of ADHD managed with psychostimulants. These children were more likely to exhibit abnormal wake systolic loads on ABPM and were less likely to be obese. While higher office BP values in children with ADHD may be related to the well known influence of psychostimulants on BP and heart rate, the prevalence of hypertension among children with ADHD has not been well studied. This study had important methodological limitation, but in view of the results, the increasing incidence of ADHD, and widespread use of psychostimulants, prospective studies aimed at defining the relationship between hypertension and ADHD in children are justified.

## Figures and Tables

**Table 1:** Patient characteristics and ABPM results.

	Patients with history of ADHD	Patients without history of ADHD	*P* value
N* (% of total)	48 (21%)	179 (79%)	
Mean age	13.02 (SD = 3.00)	12.41 (SD = 3.69)	NS^†^
Gender	M/F 40/8	M/F 100/79	*P* = 0.0009
Mean BMI	22.0 (SD = 7.48)	22.7 (SD = 6.43)	NS
Mean BMI *z*-score	0.18 (SD = 1.59)	0.75 (SD = 1.25)	*P* = 0.0089
*N* with mean systolic BP > 90%	19 (40.0%)	55 (30.7%)	NS
*N* with mean diastolic BP > 90%	5 (10.4%)	14 (7.8%)	NS
*N* with mean systolic BP > 95%	13 (27.1%)	40 (22.3%)	NS
*N* with mean diastolic BP > 95%	1 (2.1%)	8 (4.5%)	NS
*N* with mean wake systolic load > 25%	18 (37.5%)	7 (3.9%)	*P* < 0.0001
*N* with mean sleep systolic load > 25%	3 (6.3%)	45 (25.1%)	*P* = 0.0158
*N* with mean wake diastolic load > 25%	17 (35.4%)	44 (24.6%)	NS
*N* with mean sleep diastolic load > 25%	10 (20.8%)	29 (16.2%)	NS
*N* with any ABPM test abnormality	22 (45.8%)	71 (40.0%)	NS

*N: number of patients; ^†^NS: nonsignificant.
